# Surgical Approaches to Neuroblastoma: Review of the Operative Techniques

**DOI:** 10.3390/children8060446

**Published:** 2021-05-25

**Authors:** Federica Fati, Rebecca Pulvirenti, Irene Paraboschi, Giuseppe Martucciello

**Affiliations:** 1 Pediatric Surgery Unit, Women’s and Children’s Health Department, University Hospital of Padua, 35128 Padua, Italy; fedes.fati@gmail.com; 2 Dipartimento di Neuroscienze, Riabilitazione, Oftalmologia, Genetica e Scienze Materno-Infantili (DINOGMI), University of Genoa, 16147 Genoa, Italy; i.paraboschi@ucl.ac.uk (I.P.); martucciello@yahoo.com (G.M.); 3 Department of Paediatric Surgery, IRCCS Giannina Gaslini Children’s Hospital, 16147 Genoa, Italy

**Keywords:** neuroblastoma, surgery, thoracotomy, laparotomy, innovative techniques, laparoscopy, thoracoscopy

## Abstract

Neuroblastoma (NB) is the most commonly occurring soft-tissue malignancy of childhood. Surgery plays an important role in multidisciplinary treatment and its principal aim is a local control of the disease, respecting the integrity of the surrounding structures. There is no unanimous consensus on the best surgical technique, and the operative approach largely depends on the anatomical location and the extension of the mass. To have a complete overview of the different type of treatment, we made a review of the literature from the last twenty years of all the surgical approaches applied for NBs resection, accordingly to the anatomical site.

## 1. Introduction

Neuroblastoma (NB) is the most commonly occurring soft-tissue malignancy of childhood and the third most common pediatric solid malignancy after central nervous system tumors. Tumors’ treatment must always pass through a multidisciplinary approach, in which surgery is almost constantly involved. However, when facing complex neoplastic masses, it might be difficult to define the surgeon’s course of action, carefully bearing in mind the balance between risks and benefits.

At the moment there is no unanimous consensus neither on the best surgical technique and the extension of surgical resection or the right surgery timing for this disease, as it depends on the location and nature of the mass. In literature there is an ongoing debate on surgical resection and its impact on overall outcome. The main goal of surgery in patients with NB would be a most complete resection of the mass (achieving >90% resection), respecting its integrity, and avoiding excessive invasiveness and damages to the surrounding organs and tissues. The assessment of operability must take into account the feasibility of complete resection and the risk of injury to related structures; individual case discussion should lead to define the best surgical approach.

In the case of high-risk NB, multiple studies have tried to evaluate the correlation between degree of resection and outcome, in term of overall survival, through variables such as metastatic response, impact of known biological risk factors (e.g., MYCN status) and evaluation of Image Defined Risk Factors (IDRFs) at diagnosis and/or time of surgery. As a result no correlation between surgical resection and metastatic response or residual metastatic disease has been found [1].

IDRFs are useful indicators for predicting surgical risk and extent of primary resection. Anyhow, new reports suggest that not all IDRFs have the same value and utility during the planning of NB surgery. Intraspinal tumor extension, trachea compression, and main artery encasement may have a stronger influence on surgical outcomes and event-free survival [1,2].

Another aspect being discussed is the optimal timing for surgical resection in high-risk NB, which has not been well defined and is usually based on individual expertise of oncologists and surgeons. Studies show that surgery is generally feasible in the early cycles of induction chemotherapy (second or third cycle), but the impact of pre-operative treatment on operative complexity and survival remains unclear [3].

Current treatment protocols for NB consist of a combination of intensive multi-agent induction chemotherapy, surgery, radiation, myeloablative consolidation therapy with stem cell rescue and transplantation, 13-cis retinoic acid and immunotherapy [4]. This work aims to provide an overview of all the surgical treatments available for NB. To comply with our aim we made a review of the literature from the last twenty years of all the surgical approaches applied for NBs resection, according to the anatomical site.

## 2. Materials and Methods

We retrospectively reviewed the literature on NBs of the last twenty years (2000–2020). A Medline research was made and the main NBs localization (cervical, mediastinal, cervico-thoracic, thoracic, adrenal, abdominal and pelvic) were cross-matched with five different terms to capture all the existing evidence on NBs surgery. These terms were: surgery, surgical approach, surgical technique, surgical treatment, minimally invasive surgery. The yielded articles were selected based on Title and Abstract. Selection criteria were: English written articles, specified NBs site and presence of the operative technique description. The selected articles were analyzed and the different surgical approaches, depending on the tumor localization, were summarized.

## 3. Results and Discussion

### 3.1. Cervico-Thoracic Tumors

#### 3.1.1. Open Surgery

Thoracic neuroblastomas account for up to 15% of all neuroblastomas and are frequently associated with a better outcome compared to abdominal NBs [5]. According to the extension and nature of the mass, it is fundamental to choose the most appropriate surgical technique, keeping in mind that the child thorax is thinner and more elastic, allowing a bigger retraction in order to easily access the superior mediastinum [6].

Cervical NBs, with no extension to the thorax, can be removed using a cervical approach with a transverse skin incision, just above the clavicle. One the other hand, two main approaches are described in cases of mass’ extension to the thoracic inlet: the trans manubrial osteo muscular-sparing technique and the trap-door-approach (TDA) [5].

In the trans manubrial osteo muscular-sparing technique, widespread by Grunenwald, the incision extends from the thyroid cartilage, downward along the anterior margin of the sternocleidomastoid muscle, to the manubrium and the upper sternum. It is then prolonged with an anterior thoracotomy at the level of the second rib [7,8]. This approach allows a good control of the supra-aortic vessels and nerves through the thoracic inlet and a safe resection of the neoplastic mass [9,10].

The trap-door approach (TDA) is characterized by a supraclavicular incision, followed by a partial median sternotomy and associated with a transverse incision on the third or fourth intercostal space. This approach provides a good exposure of the operatory field, allowing an easy access to the neurovascular structures and a sub-adventitial dissection, when vascular encasement is present [11,12]. Different studies compare these two approaches and demonstrate that they are similar in terms complications’ rate and outcomes. The trans manubrial approach is preferred for tumors which do not extend over the posterior mediastinum (in particular those arising from the stellate ganglion). On the opposite, the trap door is preferred for tumors located in the upper part of the thorax [13,14,15,16]. Due to the close relation with the brachial plexus, Horner syndrome has often been considered an expected consequence in the cervicothoracic tumors, not related to the technique and not preventable [12,17].

A recent new approach has been proposed for the resection of cervicothoracic neuroblastomas, named Cervico-Parasternal Thoracotomy. It is characterized by an incision made along the anterior margin of the sternocleidomastoid muscle until its sternal insertion, and then vertically following the ipsilateral parasternal line. The major pectoralis muscle is detached, and the clavicle and the ribs are disarticulated from their sternal insertions. This technique allows for good exposure of the posterior costovertebral space starting from the retro-clavicular space, permitting an accurate isolation of the major subclavian blood vessels and the brachial plexus roots in order to completely expose and safely resect the mass. This approach has been applied in a limited number of patients and further follow-up is needed [18].

**Figure 1:**
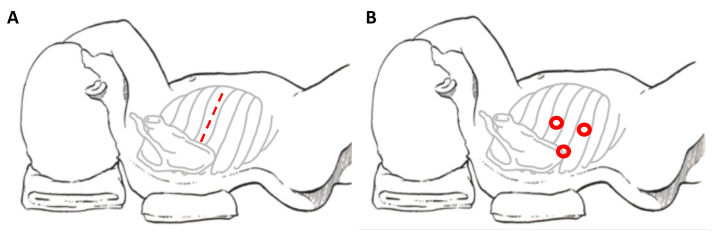
Patient’s position and site of incision in thoracotomy (**A**) and thoracoscopy (**B**), respectively.

When referring to thoracic NBs, the main traditional approach is posterolateral thoracotomy, which provides good access to the mediastinum (Figure 1A). Disadvantages of this approach include the risk of dysfunctions of ipsilateral shoulder and arm, of compromising the pulmonary function and of chronic post-thoracotomy pain syndromes [8]. In order to avoid these sequelae, minimally invasive surgery is emerging for these tumors.

#### 3.1.2. Thoracoscopy

Thoracoscopic surgery is well established as the preferred approach for many diseases involving the chest but continues to be controversial for thoracic pediatric malignancy [19]. Minimally invasive surgery (MIS) is highly effective for establishing tissue diagnosis, disease staging and assessing feasibility of complete resection in children with cancer [20]. An increasing number of retrospective reports describes a potential role of MIS in the management of different pediatric oncological entities. In fact, potential advantages in the MIS group compared to open surgery are well described, such as less intra-operative blood loss, decreased time of thoracic drainage, less pain and shorter hospital stay. All these studies are retrospective and there is a lack of prospective randomized trails assessing MIS [20,21].

Technical challenges of MIS for thoracic tumors are relevant. The use of single lung ventilation is generally recommended; however, its realization can be difficult in small children and infants [22]. Large series of thoracoscopic resection of mediastinal masses in infants highlight the anesthesiologist challenges employing single lung ventilation by mainstem intubation or double-lumen endotracheal catheter, as well as a tendency to develop a slight tension pneumothorax [23,24]. Another challenging aspect is related with the handling and the removal of the resected specimen from the thoracic cavity, as it should always be performed using a retrieval bag to avoid tumor dissemination. In some cases, for large tumor, a mini-thoracotomy might be necessary for this purpose [22], reducing the advantages of MIS. There is no absolute contraindication for VATS resection in children with thoracic neurogenic tumor. Relative contraindications can be anatomical or physiologic. Generally, this technique is preferred for neurogenic small tumors, but recent reports assert that the size of the mass is not a contraindication for thoracoscopic gross total resection.

In the case of spinal cord infiltration or tumors located very close to the vessels, it is important to have a low threshold of conversion to open approach. On the other hand, thoracic neuroblastoma has a better prognosis than extra-thoracic tumors, so that incomplete resection of the mass (more than 90%) is acceptable and visual magnification of the spinal foramina afforded by thoracoscopy allows for better control of the intercostal arteries and easy access to the thoracic paravertebral sympathetic chain, especially to the inframediastinal space [25,26,27]. When no extension of the tumor into the spinal cord is present, its complete resection is possible [25,26,28].

The development of smaller surgical instruments and the improvement of surgical techniques has helped to achieve better results for complex thoracoscopic procedures [28].

The potential advantages for the thoracoscopic approach (Figure 1B) are: better cosmetic results and lower co-morbidities compared to the lateral thoracotomy (chest wall deformities, winged scapula and scoliosis, lower duration of chest tube and shorter length of hospital stay) [22,24,26].

In children with cancer, these advantages—in particular the cosmetic outcome—take secondary priority because the goal is to achieve a complete resection of the neoplastic mass respecting the oncological rules. In this regard, one peculiar disadvantage of thoracoscopic surgery is the inability for the surgeon to directly handle the tumor; in fact, the tactile sensation, especially for highly infiltrative masses, can help surgeons to define safe tissue from the tumor itself [26]. Another limitation is the lack of proper instruments to handle the mass safely.

The most common complications are similar to thoracotomy, including chylothorax commonly linked to the location of intrathoracic neurogenic tumors (especially those in the upper thorax, near the sympathetic chain), rather than to the type of surgical resection.

Recent data suggest that port site recurrences are extremely rare and should not be a deterrent to the use of MIS [20]. A resume of the main surgical approaches for thoracic NBs can be found in Table 1.

### 3.2. Adrenal Tumors

#### 3.2.1. Open Surgery

**Figure 2:**
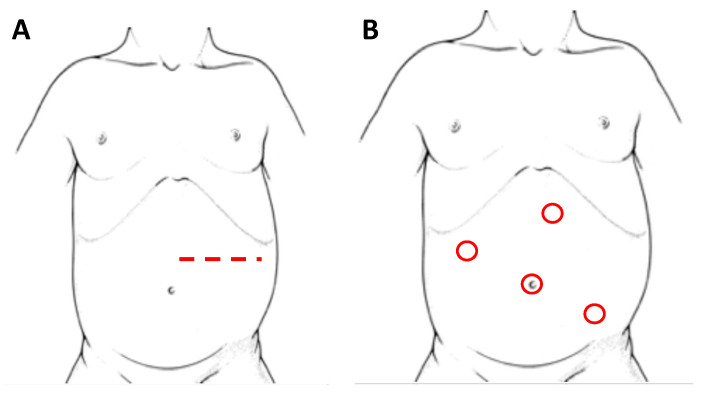
Patient’s position and site of incision/trocars in laparotomy (**A**) and laparoscopy (**B**), respectively, for left adrenal NBs.

Adrenal glands represent the main localization of abdominal NBs. In the pediatric population, most of the adrenal tumors are malignant and NBs cover up to the 85% of all the adrenal masses [30]. Historically, the preferred surgical technique consisted in an open trans-peritoneal approach through a large transverse incision (Figure 2A). Considering the infiltrative nature of these tumors, this technique remains a solid approach in order to reach a complete excision of the masses, especially in cases of vessels or lymph nodes infiltration and midline overpassing lesions.

#### 3.2.2. Minimally Invasive Surgery

With the advent of laparoscopy a growing interest in a minimally invasive technique was reported and, since its first use description by Gagner in 1992 [31], it became the gold standard for adrenal resection. The adrenal glands are particularly suitable for this kind of approach thanks to their retroperitoneal location and their small size. Just as for other abdominal interventions, laparoscopy quickly became an interesting option for treatment of NBs and the advantages that this approach can offer, compared to standard surgery, are well described in the literature. The benefits of MIS are well known, such as: the possibility of making smaller incisions, which comes along with a better cosmesis, shorter post-operative course and pain, quicker return to regular activities and less tissue trauma leading to fewer post-operative complications [32]. Also, it has been reported how laparoscopy can bring to early post-operative feeding, less bowel adhesion formation, fewer wound complications and provide some immunologic advantages that might bring to an earlier initiation of adjuvant chemotherapy. Lastly, this technique provides a wider and better view of the operative field.

The main concerns on its use are referred to the risk of incomplete resection, especially for large lesions or high-risk tumors, and the necessity of a quite long learning curve. Despite no clear guidelines on MIS, some selection criteria have been proposed, such as absence of major vascular structures encasement or IDFRs and tumor confined to the organ of origin (INSS stage 1 or 2) [33]. Although in recent years the feasibility of laparoscopic surgery seems to be wider, this technique is usually preferred for limited-size, localized and well-circumscribed lesions [34]. Tumor size does not strictly define the amenability of MIS; nonetheless most literature reports refer to tumors smaller than 4 cm [35]. Current indications, based on the International Neuroblastoma Risk Group Staging System (INRGSS) [36], see MIS as the preferred choice of treatment for adrenal localized NBs; in cases of advanced disease with large and disseminated tumor or presence of at least one pre-operative IDFR, traditional open surgery is recommended. Larger clinical studies would be necessary to define accurate indications for MIS in these patients [25].

Initially, with laparoscopy, a transperitoneal approach (Figure 2B) was performed for adrenal resection. In recent years changes in this approach have been proposed, including a retroperitoneal resection [37], a lateral laparoscopic adrenalectomy and, along with technological innovation, a robot-assisted surgery.

**Figure 3:**
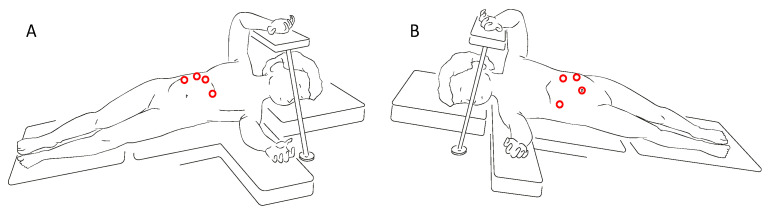
Trocars site for right (**A**) and left (**B**) lateral laparoscopic adrenalectomy.

**Figure 4:**
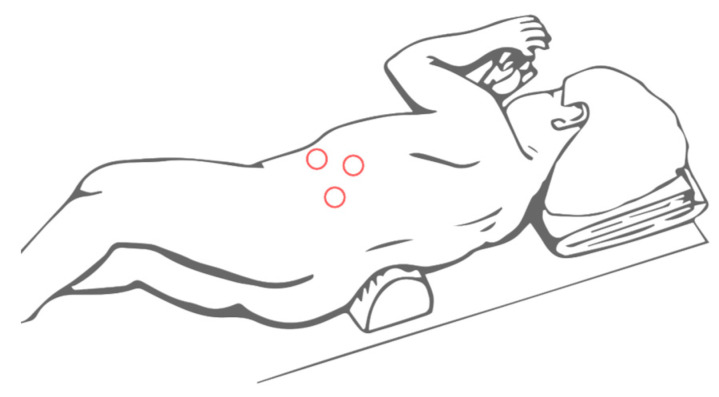
Trocars site for retroperitoneal laparoscopic adrenalectomy.

Referring to the lateral laparoscopic adrenalectomy, Catellani et al [38] describe a reverse Trendelenburg semi lateral decubitus position placement of the patients, from which a 30° optical device and the operative trocars are inserted (Figure 3). In this way a good view of the abdominal cavity and excellent exposure of the adrenal gland along with the surrounding structures are provided. It also proved to be a quickly and low-rate conversion approach, even though is preferred for small size tumors. Referring to this technique, however, some authors highlight the risk of tumoral spillage and trocar site tumoral recurrence [30]. In cases of small size tumors and absence of vessels or lymph nodes localization, retroperitoneal laparoscopic approach (Figure 4) seems to be a safe and effective alternative. It provides a great view of the adrenal glands while avoiding the dissection of the intraperitoneal structures. This is clearly associated with less abdominal adhesions formation and better bleeding control thanks to the fixed volume of the retroperitoneal space [39]. Also, a lower rate of wound dehiscence is described [40].

Robot-assisted adrenalectomy has been reported for NB surgery [41]. Characteristic small age of patients with NB makes this surgical approach poorly suitable in this setting. A resume of the surgical approaches for pediatric adrenalectomy can be found in Table 2.

Referring to MIS, another point of discussion is the correlation between tissue CO2 exposure during pneumoperitoneum and tumor dissemination. Several studies have highlighted how hypoxemia and a lower pH could improve angiogenesis and Myc (dedifferentiating factor and negative prognostic marker) over-expression in NBs cells [55]. On the other hand, Montalto et al. [56] demonstrated how, despite an initial increase of HIF-1 alpha and Myc expression after CO2 exposal, they returned at a basal level after 24 h of standard condition. Further studies should clarify whether CO2 exposure, and therefore MIS, plays a role in tumors growth and metastasis process, influencing the patient’s prognosis.

#### 3.2.3. Adrenal-Sparing Surgery

Adrenal NBs are more likely to be unilateral and the recommended treatment is always total adrenalectomy. In cases of bilateral tumor, a rare entity covering 2% of all childhood NBs, an open adrenal sparing surgery has been described with good outcome [57]. This technique feasibility mostly depends on the presence of some normal-looking adrenal tissue and the chance to preserve at least one vein and one artery. Good oncological outcomes achieved with this approach might lead to eventually consider this technique for unilateral masses as well.

### 3.3. Abdominal Tumors

While laparoscopic resection for adrenal NBs is widely performed and reported in the literature, studies on abdominal NB resection are poorly represented. In these tumors the resection of the primary mass and the lymph nodes highly correlates with a proper intra-operative visualization of the para-aortic and retroperitoneal structures [58].

At the time of surgical resection most abdominal NB are deeply connected with the surrounding structures and the surgical approach must enable the surgeon to display all the major vessels and noble structures of the abdominal cavity. For this reason an open approach is widely preferred.

In the case of spinal infiltration of the mass a sub-specialty surgical team might be required.

Direct invasion of the renal parenchyma is detected in 11 to 14% of patients and the renal vasculature is encased or narrowed in 32 to 45% of patients. Kidney-sparing surgery is widely preferred in NB surgical resection, as long as gross total resection is feasible [59]. In a recent study by Fahy et al. [60] no significant differences were observed in the survival of patients undergone kidney-sparing surgery, but a better mid-term renal function was revealed in these patients compared to the patients underwent complete nephrectomy. Taking into account the nephrotoxicity of the adjuvant treatments for NB a kidney-sparing approach is recommended.

Recent studies reveal that vessel tumor infiltration usually does not go beyond the tunaca adventitia; based on this data Kiely [61] propose an open surgery and excision of the lesion through a plane dissection between the tumor and the tunica media of the main abdominal vessels. Thanks to this technique, a complete or near complete (great than 95%) excision of the tumor was made in the majority of cases and a survival advantage of patients with stage 1–3 disease, following the International Neuroblastoma Staging System (INSS), was noted. Anyhow, vessel wall invasion is a central challenge in NB surgery, as it occurs in approximately 10% of the midline NBs. This needs to be considered in the perioperative period, as it may result in a longer operative time and a higher risk of blood losses.

A laparoscopic approach is still debated for abdominal NB, as it comes with some procedure-related technical challenges and risks, with tumor dissemination and port-site recurrence above all. Pre-operative IDRFs play an important role towards the operational technique choice; IDFRs-negative tumors are eligible for MIS.

### 3.4. Pelvic Tumors

Pelvic NB is a rare entity, covering 2 to 5% of all pediatric NB, but its surgical treatment can be challenging due to closeness of the primary mass to noble nervous structures, leading to potentially invalidating sequelae, and to a high risk of bladder or colon intra-operative damage. Prevalence of neurovascular complications after pelvic NB resection ranges between 15 to 35%. Neurologic impairment includes complete or partial cauda equine syndrome, neuropathic bladder, nerve palsy, leg weakness and long-term fecal and urinary incontinence. The standard approach for pelvic lesions is a lower median laparotomic incision, associated with neurosurgical decompression in cases of spinal involvement. Neuromuscular stimulation is usually intra-operatively required. The impact of IDRFs on the outcome of pelvic NB has been evaluated and the presence of one or more of these risk factors seems to relate with perioperative complications, local recurrence and worse oncological outcome [62].

Alternative surgical approaches common to other pelvic diseases (e.g., rhabdomyosarcomas) or congenital anomalies (e.g., ano-rectal malformations) has been described, such as retro-pubic, trans-perineal and anterior trans pubic; no major differences are reported between these techniques in terms of outcome. In recent years a new approach for pelvic lesions removal has been described (see Section 3.5).

### 3.5. New Surgical Approaches

While oncological surgery is moving toward a minimally invasive approach, some tumors may require a more invasive treatment to obtain complete exposure and control of the mass, in particular when it involves more than one anatomical compartment or when tumors are localized in deep anatomical spaces and/or in case of bilateral involvement. These conditions are not manageable by a traditional approach and may require different or combined incisions, especially when it is not possible to minimize the size of the tumor with neo-adjuvant chemotherapy. In current literature there is a paucity of indications for such lesions, and consequently the choice of surgical treatment is mainly based on the surgeon’s skills, habits and preferences. Recently, new surgical approaches have been proposed that could result in a wider all-round view of the surgical area and a radical and riskless resection. Two of the latest surgical techniques proposed are the Thoracophrenolaparotomic approach (TPL) in thoraco-abdominal tumors and the Complete Posterior Sagittal Anorectal Mobilization (PSAM) in pelvic-perineal tumors. These approaches have proved to be safe and well accepted in infants and children, with no added morbidity, and providing a better prognosis.

**Figure 5:**
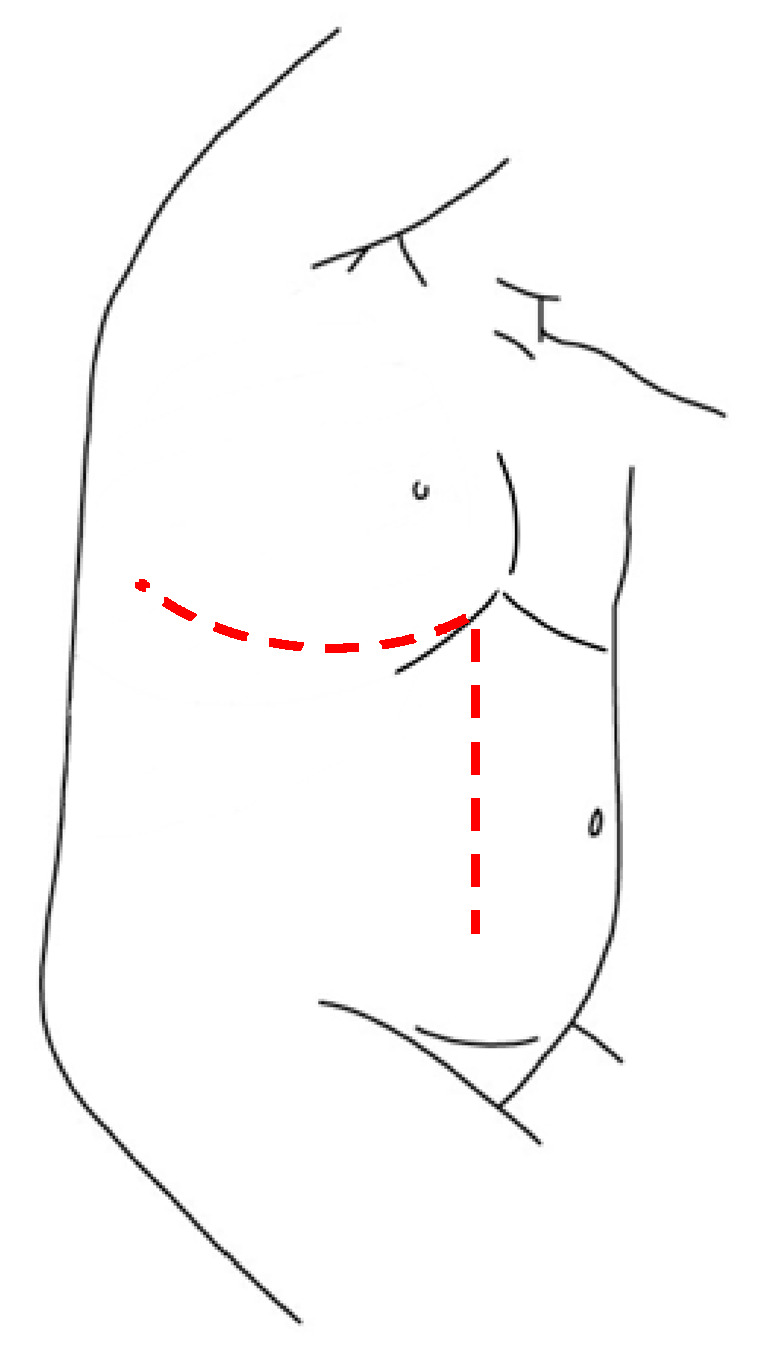
Thoracophrenolaparotomic incision for thoraco-abdominal NBs.

TPL is a well described technique in the surgical treatment of severe scoliosis in the pediatric field and of tumor’s excision in adult oncology. In literature, there are only two reports that describe the application of this surgical approach for thoraco-abdominal NBs’ treatment, proving its efficacy for gross total resection of these tumors. In this technique, the incision is made following the 10th rib, starting posteriorly from the inferior margin of the scapula and proceeding obliquely downward on the lateral margin of the *rectus abdominis* muscle beyond the umbilical transverse line. The procedure is characterized by thoracotomy, in which the periosteum-sparing partial resection of rib can is fundamental to provide a wide exposition and guarantee a regrowth of the rib, laparotomy and radial incision of the diaphragm along the posterior peripheral margin. This technique provides an excellent and complete vision of the mass, allowing a macroscopic radical tumor resection, without major complications (Figure 5) [63].

**Figure 6:**
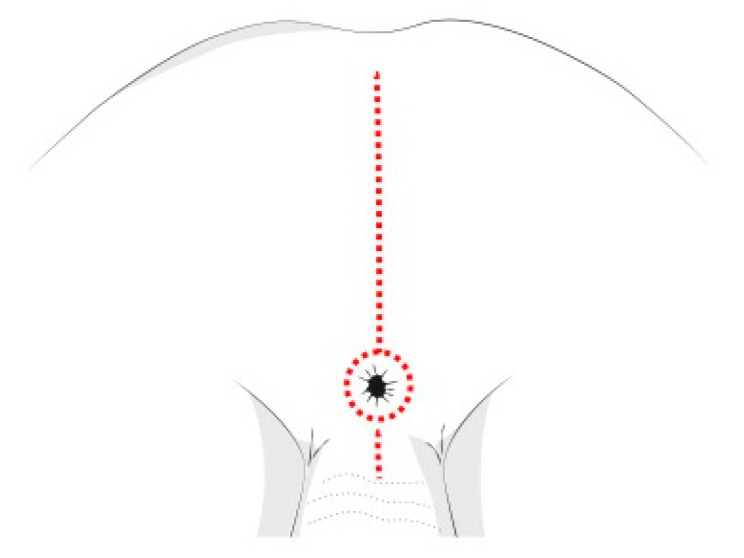
Posterior sagittal incision for pelvic NBs.

The PSAM technique follows the same principles of the Posterior Sagittal AnoRectoPlasty described by deVries and Peña [64], and routinely used for the treatment of anorectal malformations (Figure 6). This tecnique allows a complete mobilization of the rectum, granting access to the deep pelvis and enabling the surgeon to perform an accurate dissection of the tumor mass from the urethra, the bladder neck, the rectum, and other pelvic structures. This approach also reduces possible complications and sequelae of the rectal wall, like fistulas, abscesses, intrinsic wall scars and stenosis. Cosmetic results are better compared to other approaches [65].

## 4. Conclusions

Surgery plays an important role in the multidisciplinary treatment of pediatric oncologic patients, where a complete and safe resection still represents a surgical challenge.

Referring to NBs, it is still difficult to define the gold standard for surgical treatment. The operational approach largely depends on surgeon’s expertise and confidence with a technique. What can be highlighted from this review is that no proposed treatment seems to be significantly more effective than others, according to the anatomical localization of the tumor. No significant differences on complications or survival rate can be found, although some new surgical techniques provide a better exposure of the operative field and determine functional and aesthetic outcome differences.

In the current literature several studies comparing traditional open surgery and MIS can be found; cosmetic and post-operative pain advantages of a mini-invasive approach are well defined, even in the pediatric field. Regardless of surgical innovation, defined guidelines on MIS usage in oncologic patients are lacking and more comparative studies between the existing approaches for same-site NBs would be necessary. The existing evidence does not provide enough data to determine the best clinical practice for NBs, and more large, randomized clinical trials would be necessary to safely expand MIS usage and establish a standardized protocol for these tumors’ treatment, accordingly to the anatomical region involved in the disease. A multidisciplinary approach to these patients remains the key-point to obtain the higher survival rates, along with the best quality of life. Surgery’s main goal is to provide a complete excision of the masses, always considering the right balance between risks and benefits.

## Figures and Tables

**Table 1:** Comparation of surgical technique for thoracic NBs. (TS = Thoracoscopic; TT = Thoracotomy; NB = neuroblastoma; GNB = ganglio neuroblastoma; GN = ganglioneuroma; MPST = Malignant peripheral nerve sheath tumor ND = not defined).

Author/Year	*N* Patient	Age (Months)	Surgical Approach	Operative Time (Minutes)	Conversion	Stage	Type	N-Myc ampL	Hospital Stay (Days)	Post-Operative Complication	Outcome	Follow Up Months
Decou, J. 2005 [29]	5	27.2	TS	108.6	none	1	NB	no	2.6	2 spillage	alive, complete remission	29.2
Fraga, J.C. 2010 [21]	43	36	TT (*n* = 38); TS (*n* = 5)	TT 132; TS 180	none	8 stage I, 11 stage II, 8 stage III, 6 stage IV, 3 stage IVS	10 NB, 13 GNB, 10 GN	ND	TT 6; TS 4	5 Horner syndr 2 chylothorax; 2 pnuemothorax, 1 empiema, 1 tracheomalacia and paralysis of diaphragm	4 recurrences, 2 deaths	41.5
Fraga, J.C. 2012 [28]	17	16	TS	90	none	4 stage I, 5 stage II, 3 stage III, 1 stage IV-S	10 NB, 3 GNB, 4 GN		3	2 Horner syndrome	alive, complete remission	16
Irtan, S. 2014 [25]	20	39	TS	ND	2	5 L1, 5 L2, 7 M, 3 MS	3 GN, 9 NB, 8 GNB	ND	ND	1 Horner syndr, 3 chylothorax	alive, complete remission	33
Lacreuse, I. 2007 [26]	21	72	TS	100	none	ND	9 NB, 9GNB, 3 GN	no	4.5	2 chylothorax	alive, complete remission	48
Malek, M.M. 2010 [27]	37	13 (TS), 6 (TT)	TS (*n* = 11), TT (*n* = 26)	TS 150, TT 180	none	10 stage I, 18 stage II 18, 3 stage III, 4 stage IV	NB	2	TS 2, TT 3.5	2 Horner’s syndrome, 1 chylothorax, 1 postoperative scoliosis, and 1 severe atelectasis	5 recurrence, 23 free survival	ND
Nio, M. 2005 [23]	6	42	TS	230	none	ND	1 NB, 2 GNB, 3 GN,	ND	7.6	none	ND	49
Petty, J.K. 2006 [20]	17	10 (TS), 7 (TT)	TS (*n* = 10), TT (*n* = 7)	TS 54, TT 138	1	5 stage I, 3 stage II, 2 stage IV	10 NB, 5 GN, 1 MPST	ND	TS 2, TT 4	5 Horner syndr, 1 pleural effusion	1 tumour progression	19

**Table 2:** Comparison of surgical techniques for adrenalectomy. (TLLA = Transperitoneal Lateral Laparoscopic Adrenalectomy; LA = Laparoscopic Adrenalectomy; TLA = Transperitoneal Laparoscopic Adrenalectomy; RPSA = Retroperineoscopic Adrenalectomy; OA = Open Adrenalectomy; * = Open Adrenalectomy group).

Author/Year	*N* Patient	Age (Months)	Surgical Approach	Operative Time (Minutes)	Conversion	Hospital Stay (Days)	Post-Operative Complication	Outcome	Follow Up Months
Catellani et al., 2014 [38]	4	87	TLLA	85–125	0	3.75	0	Alive, no disease recurrence	35.25
Mirallié et al., 2001 [40]	6	97.34	LA	191.25	2	/	0	1 patient remained hypertensive	1
Mitra et al., 2020 [41]	3	76	Robotic-assisted LA	244	0	2	1 morbilliform eruption	Alive, no disease recurrence	19
Al-Shanafey 2008 [42]	29	36	TLA	144	3	2	0	Alive, no disease recurrence	36
De Barros et al., 2012 [30]	7	27	TLA	138.6	1	2.9	0	Alive, no disease recurrence	18.8
De Lagausie et al., 2003 [43]	9	38	LA	/	1	4.5	1	1 disease recurrence	/
Fascetti Leon et al., 2016 [32]	68	62	63 TLA 5 RPSA	227.5	/	4.5	/	2 disease recurrence	52
Kadamba et al., 2004 [44]	10	48	TLA	235.5	2	3	0	1 death for tumor dissemination, 1 patient on chemotherapic treatment	24
Kelleher et al., 2013 [45]	79	32.3	61 OA 18 LA	OA 292 LA 168.5	2	OA 10.4 LA 3.5	1 sepsis (LPT group)	23 deaths	OA 56.5 LA 30.5
Kouch et al., 2003 [46]	6	8.5	RPSA	195	0	/	/	Alive, no disease recurrence	15-29
Lopes et al., 2012 [47]	19	46.8	LA	138.5	0	3.5	0	4 disease recurrence	81
Mattioli et al., 2014 [48]	55	14	LA	90	0	4	0	Alive, no disease recurrence	27
Nerli et al., 2011 [49]	18	69.6	LA	95	0	2	0	Alive, no disease recurrence	39
Peter et al., 2011 [50]	140	105.6	LA	140.7	13	/	1 renal infarction	1 local recurrence	18
Saad et al., 2005 [51]	6	26.2	LA	149.2	0	1	0	/	21
Yao et al., 2018 [52]	37	37.24	24 OA 13 LA	OA 143.13 LA 143.85	2	2	/	2 disease recurrence *	86.78
Meignan et al., 2017 [53]	3	11.7	Robot-assisted LA	104.3	0	2.3	0	Alive, no disease recurrence	41.6
Stanford et al., 2002 [54]	64	OA 106.8 LA 168	60 OA 4 LA	OA 236 LA 264	/	OA 5.4 LA 2.7	0	2 local recurrence	/
Romano et al., 2007 [37]	26	OA 41.7 RPSA 62.4	19 OA 7 RPSA	OA 203.7 RPSA 97.1	0	/	0	3 deaths * 3 patients on chemotherapic treatment	/
